# MiR-21 mediates sorafenib resistance of hepatocellular carcinoma cells by inhibiting autophagy via the PTEN/Akt pathway

**DOI:** 10.18632/oncotarget.4814

**Published:** 2015-07-30

**Authors:** Changjun He, Xuesong Dong, Bo Zhai, Xian Jiang, Deli Dong, Baoxin Li, Hongchi Jiang, Shidong Xu, Xueying Sun

**Affiliations:** ^1^ Department of Surgery, the Affiliated Cancer Hospital of Harbin Medical University, Harbin, China; ^2^ Department of Pharmacology, the State-Province Key Laboratories of Biomedicine-Pharmaceutics of China, Harbin Medical University, Harbin, China; ^3^ The Hepatosplenic Surgery Center, Department of General Surgery, the First Affiliated Hospital of Harbin Medical University, Harbin, China

**Keywords:** sorafenib, hepatocellular carcinoma, miR-21, phosphatase and tensin homolog, Akt, autophagy

## Abstract

Sorafenib resistance remains a major obstacle for the effective treatments of hepatocellular carcinoma (HCC). Recent studies indicate that activated Akt contributes to the acquired resistance to sorafenib, and miR-21 dysregulates phosphatase and tensin homolog (PTEN), which inhibits Akt activation. Sorafenib-resistant HCC cells were shown to be refractory to sorafenib-induced growth inhibition and apoptosis. Akt and its downstream factors were highly activated and/or upregulated in sorafenib-resistant cells. Inhibition of autophagy decreased the sensitivity of sorafenib-resistant cells to sorafenib, while its induction had the opposite effect. Differential screening of miRNAs showed higher levels of miR-21 in sorafenib-resistant HCC cells. Exposure of HCC cells to sorafenib led to an increase in miR-21 expression, a decrease in PTEN expression and sequential Akt activation. Transfection of miR-21 mimics in HCC cells restored sorafenib resistance by inhibiting autophagy. Anti-miR-21 oligonucleotides re-sensitized sorafenib-resistant cells by promoting autophagy. Inhibition of miR-21 enhances the efficacy of sorafenib in treating sorafenib-resistant HCC tumors *in vivo*. We conclude that miR-21 participates in the acquired resistance of sorafenib by suppresing autophagy through the Akt/PTEN pathway. MiR-21 could serve as a therapeutic target for overcoming sorafenib resistance in the treatment of HCC.

## INTRODUCTION

Hepatocellular carcinoma (HCC) is the second leading cause of worldwide cancer-related death in men [1]. It is notoriously resistant to chemotherapy [2]. Sorafenib is a first-line systemic drug for advanced HCC, but has only limited survival benefits [3, 4]. It is, therefore, important to unravel the molecular mechanisms that underlie sorafenib resistance in HCC.

As a multi-targeted tyrosine kinase inhibitor (TKI), sorafenib inhibits the Raf/mitogen-activated protein kinase (MAPK)/extracellular signaling-regulated kinase (ERK) pathway, and several tyrosine kinase receptors [3]. The phosphatidylinositol 3-kinase (PI3K)/Akt pathway cross-talks with the Raf/MAPK/ERK pathway [5], and represents a key signaling pathway activated in hepatocarcinogenesis [6, 7]. Sorafenib activates Akt [8] and its downstream factors such as ribosomal protein S6 kinase (S6K) and eukaryotic translation initiation factor 4E-binding protein 1 (4EBP1) in HCC cells [9, 10]. Blockade of the PI3K/Akt pathway increases the sensitivity of sorafenib against HCC [9, 11, 12]. We have recently demonstrated that sorafenib-resistant HCC cells had increased expression of phosphorylated Akt (p-Akt), and inhibition of Akt reversed the acquired resistance to sorafenib [13]. However, the mechanisms by which sorafenib activates Akt in HCC remain unclear.

MicroRNAs (miRNAs) are small noncoding RNAs that act by post-transcriptional silencing. They play critical roles in regulating multiple cellular functions [14]. Among these, miRNA-21 is one of the well characterized miRNAs and is consistently overexpressed in various types of malignancies including HCC [15, 16, 17]. Overexpressed miR-21 contributes to increased cell proliferation and a decline in apoptosis by suppressing several tumor suppressor genes including phosphatase and tensin homolog (PTEN), tropomyosin 1, programmed cell death 4, maspin and metallopeptidase inhibitor 3 [15, 18]. Recent studies reveal that miR-21 induces activation of the PI3K/Akt pathway through inhibiting PTEN [17, 19, 20, 21]. MiR-21 has also been shown to be a therapeutic target for reversing drug resistance in several types of cancer [22]. For instance, it contributes to gefitinib resistance in non-small cell lung cancer [23, 24], trastuzumab resistance in breast cancer [20], and the resistance to interferon-α/5-fluorouracil in HCC [25]. However, it remains unclear whether miR-21 also mediates the acquired resistance of HCC cells to sorafenib.

Autophagy was initially regarded as a self-digestion process [26], but more recently it has been considered to be the Type II programmed cell death (PCD) [27]. It is well recognized that apoptosis is the Type I PCD and a tumor suppressing pathway, but autophagy is a double-edged sword depending on the cellular context and nature of the stimuli [28]. It has been reported that sorafenib induced autophagic death of HCC cells [29]. We have recently reported that autophagy participates in sorafenib resistance, and that inhibition of autophagy sensitizes HCC cells to sorafenib [13]. The activated Akt/ mammalian target of rapamycin (mTOR) pathway exerts an inhibitory effect on autophagy by dysregulating autophagy-related protein (ATG) 6 (also known as Beclin-1) and ATG8 (microtubule-associated protein 1 light chain 3, LC3) [13, 30, 31]. As a typical target gene of miR-21, PTEN induces strong inhibition of autophagy [32]. Targeting miR-21 confers radio-sensitivity of glioma cells [33], and increases chemosensitivity of leukemia cells [34], by enhancing autophagy. These results indicate that miR-21 may regulate sorafenib resistance through its effects on autophagy.

## RESULTS

### Sorafenib-resistant HCC cells are insensitive to sorafenib by activating the Akt/mTOR pathway

**Figure 1:**
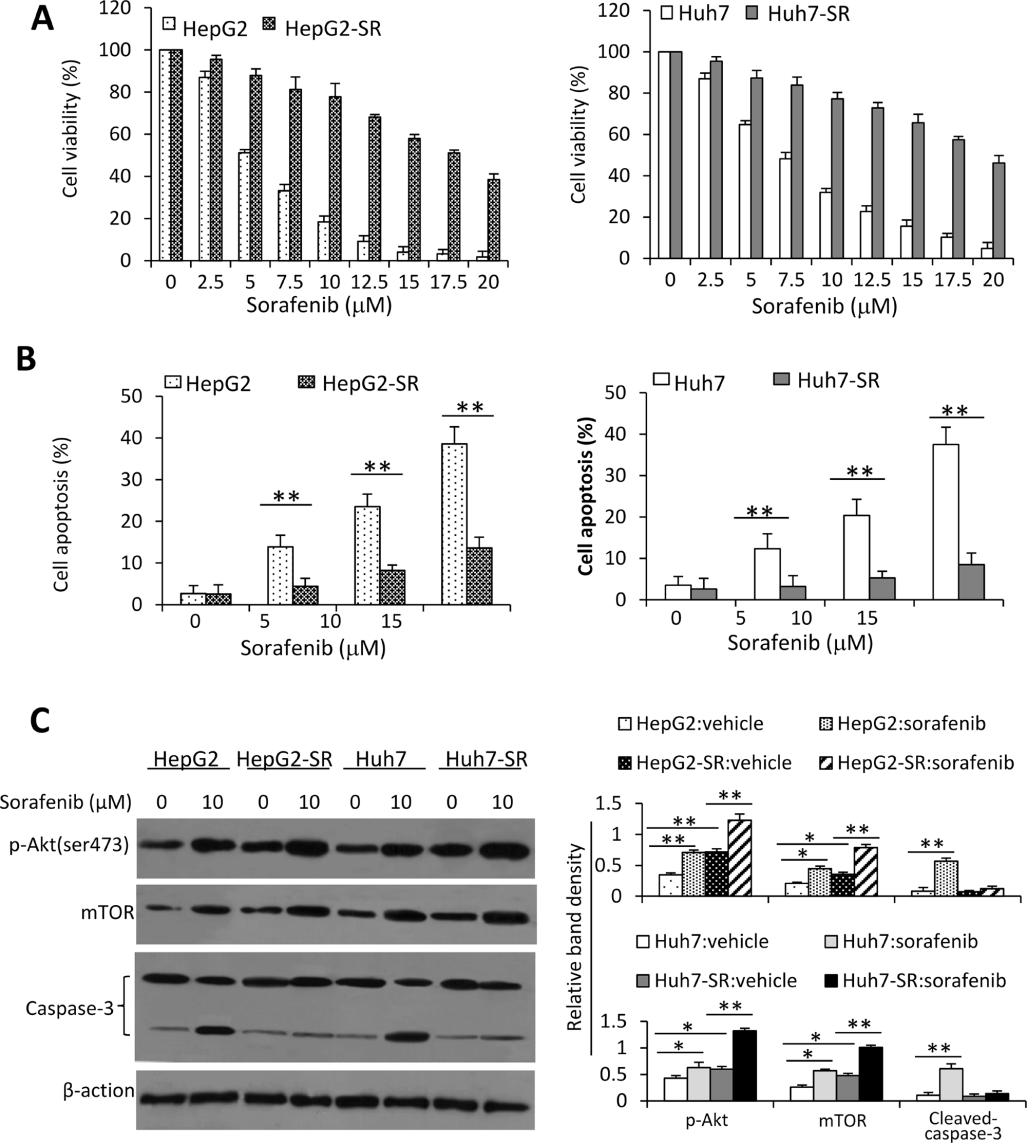
Sorafenib-resistant HCC cells are insensitive to sorafenib **A.** HepG2, HepG2-SR, Huh7 and Huh7-SR cells were incubated with serial concentrations of sorafenib for 48 h. Cell viability (%) was measured and normalized with the corresponding untreated cells. **B.** The above cells were incubated with sorafenib (0, 5, 10 or 15 μM) for 48 h, and then analyzed cytometrically to detect apoptosis. **C.** Lysates from the above cells incubated with sorafenib (0, 10 μM) were immunoblotted. **D.** The density of each band was measured and normalized to respective β-actin. “*” (*P* < 0.05) and “**” (*P* < 0.001) indicate a significant difference.

Two sorafenib-resistant cell lines, termed HepG2-SR and Huh7-SR, were established by chronic exposing HepG2 and Huh7 cells with increasing concentrations of sorafenib, respectively. Incubation of sorafenib with HepG2 and Huh cells reduced their viability in a concentration-dependent manner (Figure 1A). However, HepG2-SR and Huh7-SR cells became resistant to sorafenib as their viability was significantly higher than that of respective parental cells when exposed to the same concentration of sorafenib (Figure 1A). In the presence of 20 μM sorafenib, the viability of HepG2-SR and Huh7-SR were 36.3% and 45.7%, respectively, while the parental cells were almost completely non-viable (Figure 1A). These results are in agreement with previous reports [13, 35, 36], and are supported by apoptosis results. Thus sorafenib-resistant cells become refractory to sorafenib-induced apoptosis (Figure 1B).

We next examined alterations of the key molecules in the Akt/mTOR pathway. Sorafenib-resistant cells expressed higher levels of p-Akt, resulting in upregulation of mTOR and differential expression of other downstream factors, when compared with their respective parental cells (Figure 1C and Supplementary Figure S1). Incubation of sorafenib resulted in increased activation of Akt, leading to the increased expression of mTOR, in both parental and sorafenib-resistant cells (Figure 1C and Supplementary Figure S1). These results indicate that sustained exposure of HCC cells to sorafenib could activate the Akt/mTOR pathway.

### Inhibition of autophagy contributes to sorafenib resistance in HCC cells

**Figure 2:**
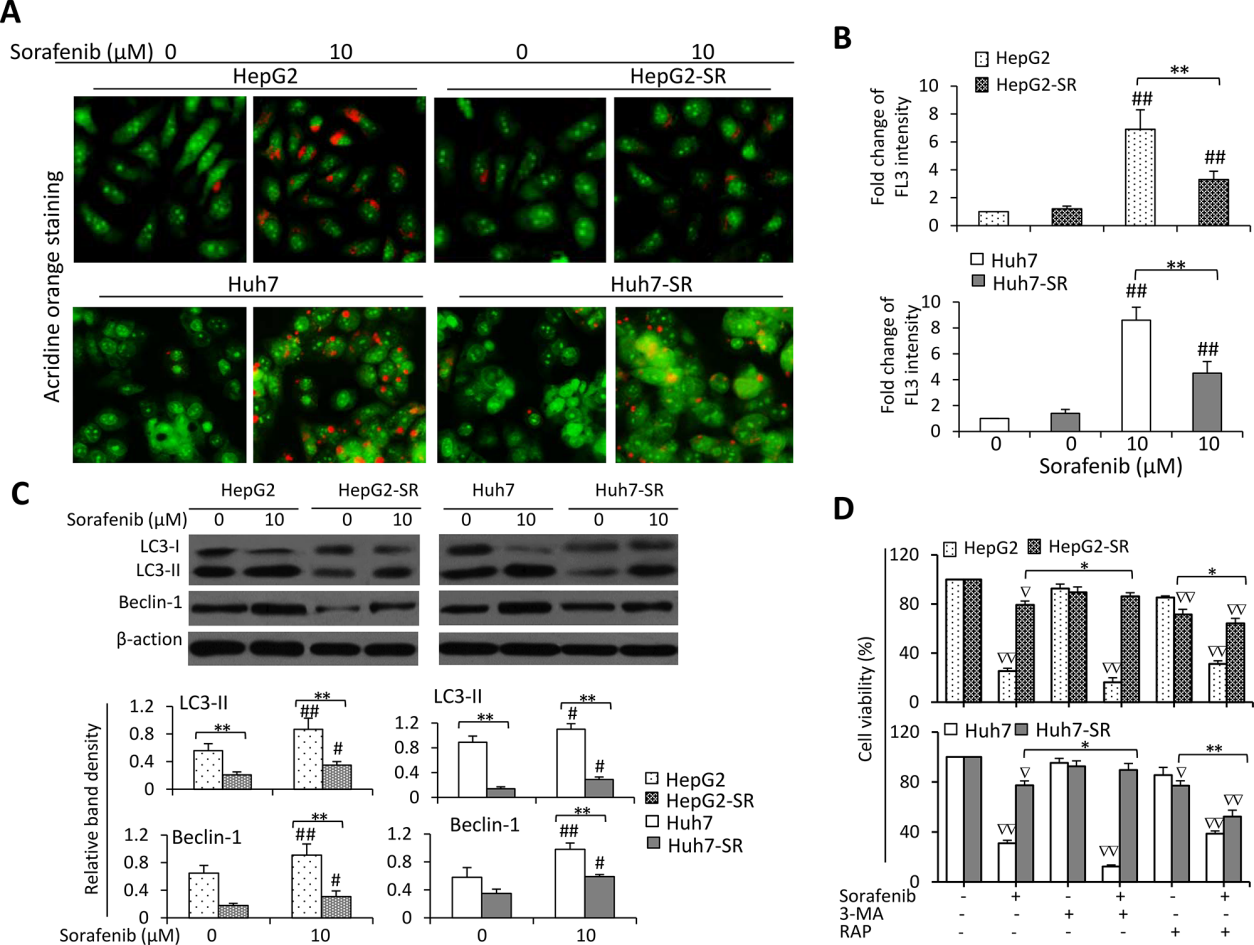
Sorafenib-resistant cells are resistant to sorafenib-induced autophagy **A.** HepG2, HepG2-SR, Huh7 or Huh7-SR cells were incubated sorafenib (0, 10 μM) for 48 h, and then stained by acridine orange. **B.** The above cells were subjected to flow cytometry, and the fold change of acridine orange fluorescence intensity (FL3) versus untreated parental cells was calculated. **C.** The above cells were immunoblotted. The density of each band was measured and normalized to respective β-actin. **D.** Cells were incubated for 48 h in the presence or absence of sorafenib (10 μM), 3-MA (3-methyladenine) (10 mM), RAP (rapamycin) (10 nM), or the combination. Cell viability (%) was compared the corresponding untreated cells. “*” (*P* < 0.05) and “**” (*P* < 0.001) indicate a significant difference. “#” (*P* < 0.05) and “##” (*P* < 0.001) indicate a significant increase; while “∇;#x201D; (*P* < 0.05) and “∇∇” (*P* < 0.001), a significant reduction, versus respective untreated cells.

Given that mTOR is a gatekeeper of autophagy by dysregulating ATGs [37], we next investigated whether inhibition of autophagy contributes to the acquired resistance to sorafenib. Incubation of sorafenib led to more acridine orange-stained acidic vesicular organelles (AVOs) in both parental and sorafenib-resistant cells, but parental cells had more AVOs than sorafenib-resistant cells (Figure 2A). Quantitative analysis confirmed that the sorafenib-resistant cells had significantly lower FL3 intensity than their parental cells in the presence of sorafenib (Figure 2B). The results were further supported by staining the above cells with monodansylcadaverine (MDC), a marker for autophagic vacuoles (Supplementary Figure S2). Immunoblotting analysis showed that sorafenib upregulated the expression of LC3-II and Beclin-1, two key autophagic proteins, in both parental and sorafenib-resistant cells; but parental cells expressed higher levels of LC3-II and Beclin-1 than sorafenib-resistant cells (Figure 2C). Suppression of autophagy by 3-methyladenine (3-MA) protected sorafenib-resistant cells against sorafenib-induced reduction in cell viability. In contrast, rapamycin (RAP), an inhibitor of mTOR and an inducer of autophagy [38], increased sorafenib-induced growth inhibition of sorafenib-resistant cells (Figure 2D). To further verify the role of autophagy in sorafenib-induced reduction in cell viability, we applied a mixture of E-64d (10 μg/ml) and pepstatin A (10 μg/ml), which are lysosomal protease inhibitors that inhibit the formation of the autophagolysosomes and late-stage autophagy. E-64d/pepstatin A attenuated sorafenib-induced reduction in cell viability in sorafenib-resistant cells. Sorafenib induced increased LC3-II expression and reduced p62 expressions [13], which were not abolished by E-64d/pepstatin A (Supplementary Figure S3).

**Figure 3:**
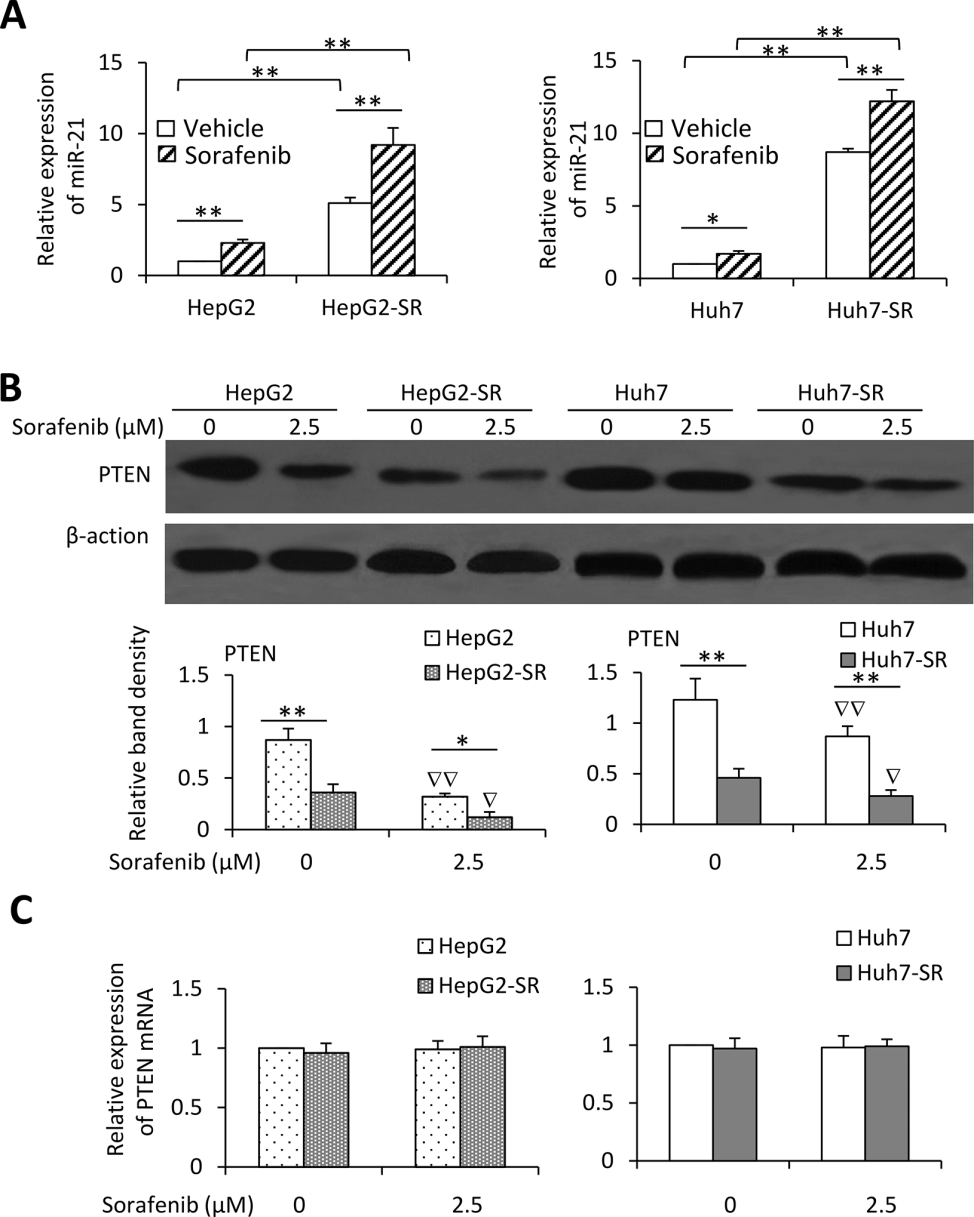
Exposure to sorafenib induces upregulation of miR-21 and downregulation of PTEN **A.** HepG2, HepG2-SR, Huh7 and Huh7-SR cells were incubated with sorafenib (2.5 μM) for 96 h, and then the expression of miR-21 was measured by real-time RT-PCR. The level of mRNA from untreated parental cells was defined as 1. **B.** The above cells were immunoblotted. The density of each band was normalized to respective β-actin. **C.** The above cells were subjected to real-time RT-PCR to measure PTEN mRNA. The relative expression level in untreated parental cells was defined as 1.0. “*” (*P* < 0.05) and “**” (*P* < 0.001) indicate a significant difference. “∇” (*P* < 0.05) and “∇∇” (*P* < 0.001) indicate a significant reduction versus respective untreated cells.

### Sorafenib-resistant HCC cells express higher levels of miR-21

We next analyzed the miRNA expression profiles in Huh7 and Huh7-SR cells by using a Human MicroRNA Array. Pairwise significance analysis of the data indicate that the expression of 16 miRNAs was significantly higher, while the expression of 8 miRNAs was significantly lower in Huh7-SR cells, compared with Huh7 cells (Supplementary Table S1). To validate the differential expression patterns of these 24 miRNAs, real-time RT-PCR was performed for the 4 most upregulated and 2 most downregulated miRNAs in HepG2, HepG2-SR, Huh7 and Huh7-SR cells (Supplementary Figure S4). We further incubated the cells with lower concentration of sorafenib (2.5 μM) for 96 h, and examined the expression of miR-21 by real-time RT-PCR. Exposure of either parental or sorafenib-resistant HCC cells to the lower concentration of sorafenib upregulated the expression of miR-21, though sorafenib-resistant cells expressed higher levels of miR-21 than their parental cells, in the presence or absence of sorafenib (Figure 3A). PTEN is a known miR-21 target gene in HCC cells [15, 17]. We confirmed that sorafenib-resistant cells expressed lower levels of PTEN protein, and sorafenib significantly reduced the expression of PTEN in both parental and sorafenib-resistant HCC cells (Figure 3B). However, the levels of PTEN mRNA remained unchanged between sorafenib-resistant and parental cells with or without sorafenib incubation (Figure 3C).

**Figure 4:**
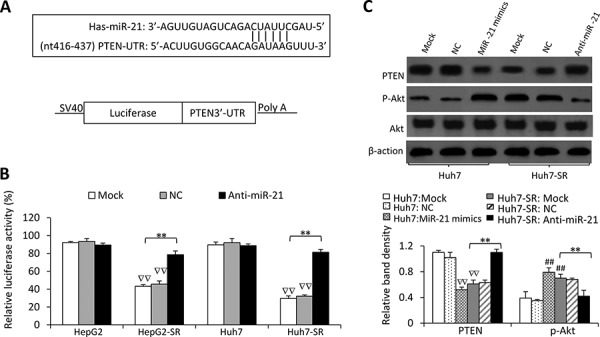
MiR-21 regulates PTEN expression and Akt activation in sorafenib-resistant cells **A.** Predicted paring of miR-21 to the 3′-UTR of the human PTEN gene, and the diagram of a pMIR-REPORT luciferase reporter vector containing the 3′-UTR of PTEN with miR-21 seed site (pMIR-luc-PTEN3′UTR-miR21). **B.** Luciferase activities in HepG2, HepG2-SR, Huh7 and Huh7-SR cells transfected with pMIR-luc-PTEN3′UTR-miR21 or a control vector. The cells were mock co-transfected or co-transfected with negative control (NC) or anti-miR-21 oligonucleotides. Relative luciferase activity was calculated as the percentage of luciferase activity in pMIR-luc-PTEN3′UTR-miR21-tranfected cells over those with the control vector. **C.** The above cells were immunoblotted. The density of each band was measured and normalized to respective β-actin. “**” (*P* < 0.001) indicates a significant difference. “##” (*P* < 0.001) indicate a significant increase; while “∇∇” (*P* < 0.001), a significant reduction, versus mock transfected parental cells.

### MiR-21 regulates PTEN expression and Akt activation in HCC cells

To investigate the function of miR-21, we transfected a luciferase reporter containing the 3′-UTR of PTEN with a miR-21 seed site (Figure 4A) into parental and sorafenib-resistant HCC cells. In agreement of miR-21 upregulation, luciferase activities were suppressed by 53.6± 2.4% and 70.1 ± 3.3% in HepG2-SR and Huh7-SR cells, respectively, whereas the suppressions were only 6.5–10% in their parental cells (Figure 4B). However, co-transfection of anti-miR-21 oligonucleotides significantly attenuated the suppression in luciferase activity in sorafenib-resistant cells, but did not significantly alter luciferase activities in parental cells. These results indicate that miR-21 overexpression in sorafenib-resistant HCC cells is accompanied by the elevated targeting function of miR-21.

We next examined whether changing miR-21 by exogenous transfection could lead to the change of PTEN protein expression and the activation of Akt, which is negatively regulated by PTEN [17]. Transfection of miR-21 mimics significantly reduced the expression of PTEN protein and increased the activation of Akt in parental HCC cells; whereas anti-miR-21 significantly increased the expression of PTEN protein and inhibited the activation of Akt in sorafenib-resistant HCC cells (Figure 4C).

### MiR-21 levels influences the effects of sorafenib on HCC cells

**Figure 5:**
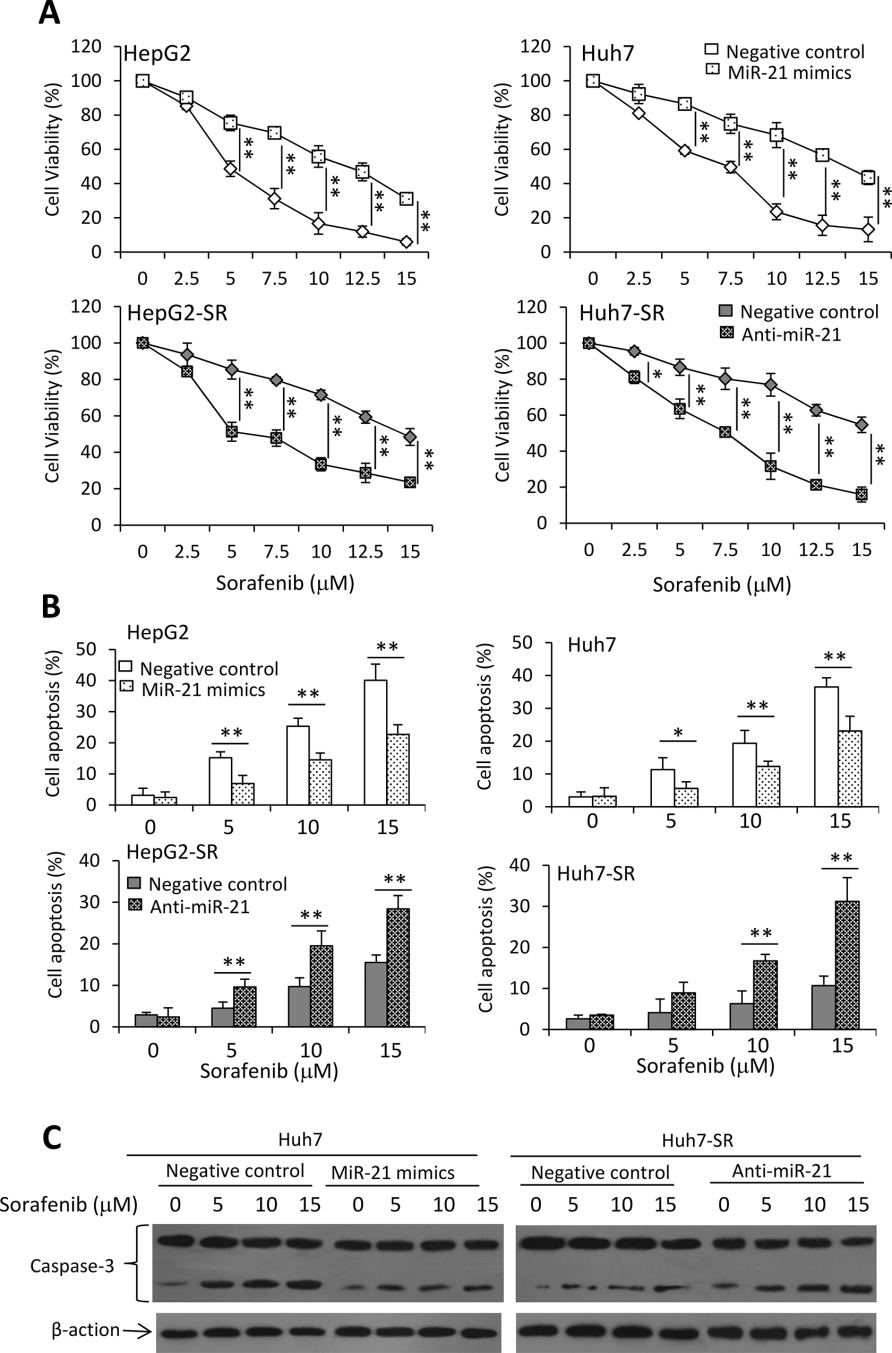
MiR-21 affects sorafenib-induced cell growth inhibition and apoptosis **A.** HepG2, HepG2-SR, Huh7 or Huh7-SR cells transfected with negative control, miR-21 mimics or anti-miR-21 oligonucleotides were incubated with serial concentrations of sorafenib for 48 h. Cell viability (%) was measured and normalized with the corresponding untreated cells. **B.** The above cells incubated with sorafenib (0, 5, 10 or 15 μM) for 48 h were analyzed cytometrically to detect apoptosis. **C.** The above Huh7 and Huh7-SR cells were immunoblotted for detecting the expression of caspase-3. “*” (*P* < 0.05) and “**” (*P* < 0.001) indicate a significant difference.

Given that miR-21 regulates PTEN expression and sequential Akt activation, which contribute to the sorafenib resistance in HCC cells, we next examined whether miR-21 could influence the effects of sorafenib on the viability and apoptosis of HCC cells. Transfection of miR-21 mimics reduced the inhibitory effects of sorafenib on the viability of parental HCC cells, while transfection of anti-miR-21 oligonucleotides enhanced the inhibitory effects of sorafenib in sorafenib-resistant HCC cells (Figure 5A). These results were supported by apoptosis assays, which showed that transfection of miR-21 mimics reduced the pro-apoptotic activity of sorafenib in parental HCC cells, while anti-miR-21 enhanced the pro-apoptotic activity of sorafenib in sorafenib-resistant cells (Figure 5B). The results were supported by the activation of caspase-3 examined by immunoblotting analysis (Figure 5C).

### MiR-21 affects sorafenib-induced autophagy in HCC cells

**Figure 6:**
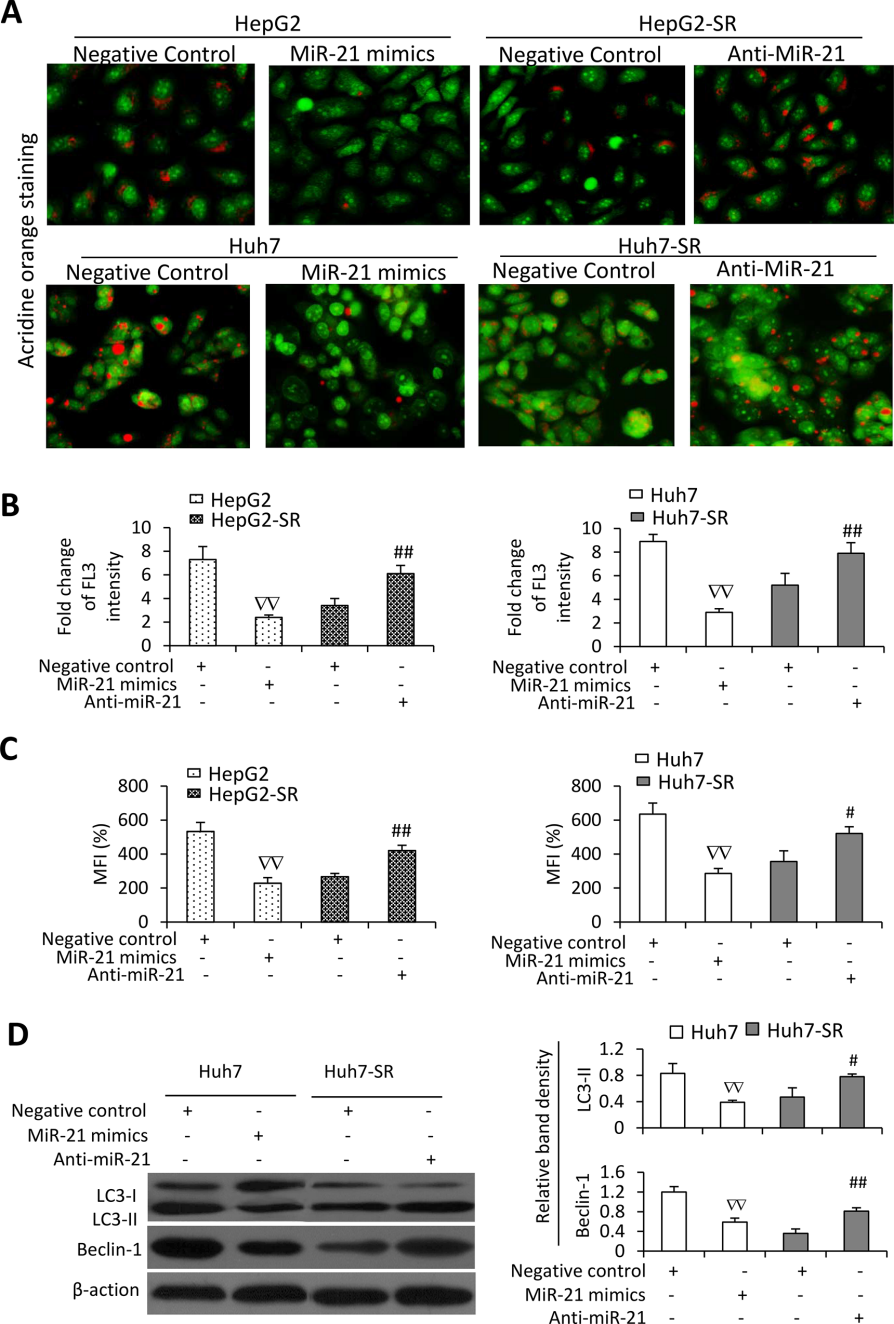
MiR-21 inhibits sorafenib-induced autophagy in HCC cells HepG2, HepG2-SR, Huh7 or Huh7-SR cells transfected with negative control, miR-21 mimics or anti-miR-21 oligonucleotides were incubated with sorafenib (10 μM) for 48 h. **A.** Representative images were taken from acridine orange-stained cells. **B.** The above cells were subjected to flow cytometry, and the fold change of acridine orange fluorescence intensity (FL3) versus untreated parental cells was calculated. **C.** The above cells were stained with monodansycadaverine (MDC) and the mean fluorescence intensity (MFI) (% of control) was measured by flow cytometry. Untreated parental cells served as controls. **D.** The above cells were immunoblotted. The density of each band was measured and normalized to respective β-actin. “#” (*P* < 0.05) and “##” (*P* < 0.001) indicate a significant increase; while “∇∇” (*P* < 0.001), a significant reduction, versus respective negative control-transfected cells.

Since autophagy participates in sorafenib resistance in HCC cells [13], we next investigated the effects of miR-21 on autophagy of HCC cells. MiR-21 mimics inhibited sorafenib-induced autophagy in HepG2 and Huh7 cells since fewer acridine orange-stained AVOs were observed in miR-21 mimics-transfected cells than in control oligonucleotides-transfected cells (Figure 6A). On the other hand, transfection of anti-miR-21 oligonucleotides increased autophagy of HepG2-SR and Huh7-SR cells induced by sorafenib since anti-miR-21-transfected cells had more acridine orange-stained AVOs than control oligonucleotides-transfected cells (Figure 6A). These results were in agreement with quantitative analysis by flow cytometry (Figure 6B). The results of acridine orange assay were supported by the data from MDC staining (Figure 6C) and immunoblotting data (Figure 6D). MiR-21 mimics decreased the expression of LC3-II and Beclin-1 in Huh7 cells, while anti-miR-21 increased their expression in Huh7-SR cells (Figure 6D).

### Inhibition of miR-21 enhances the efficacy of sorafenib in treating sorafenib-resistant HCC tumors *in vivo*

**Figure 7:**
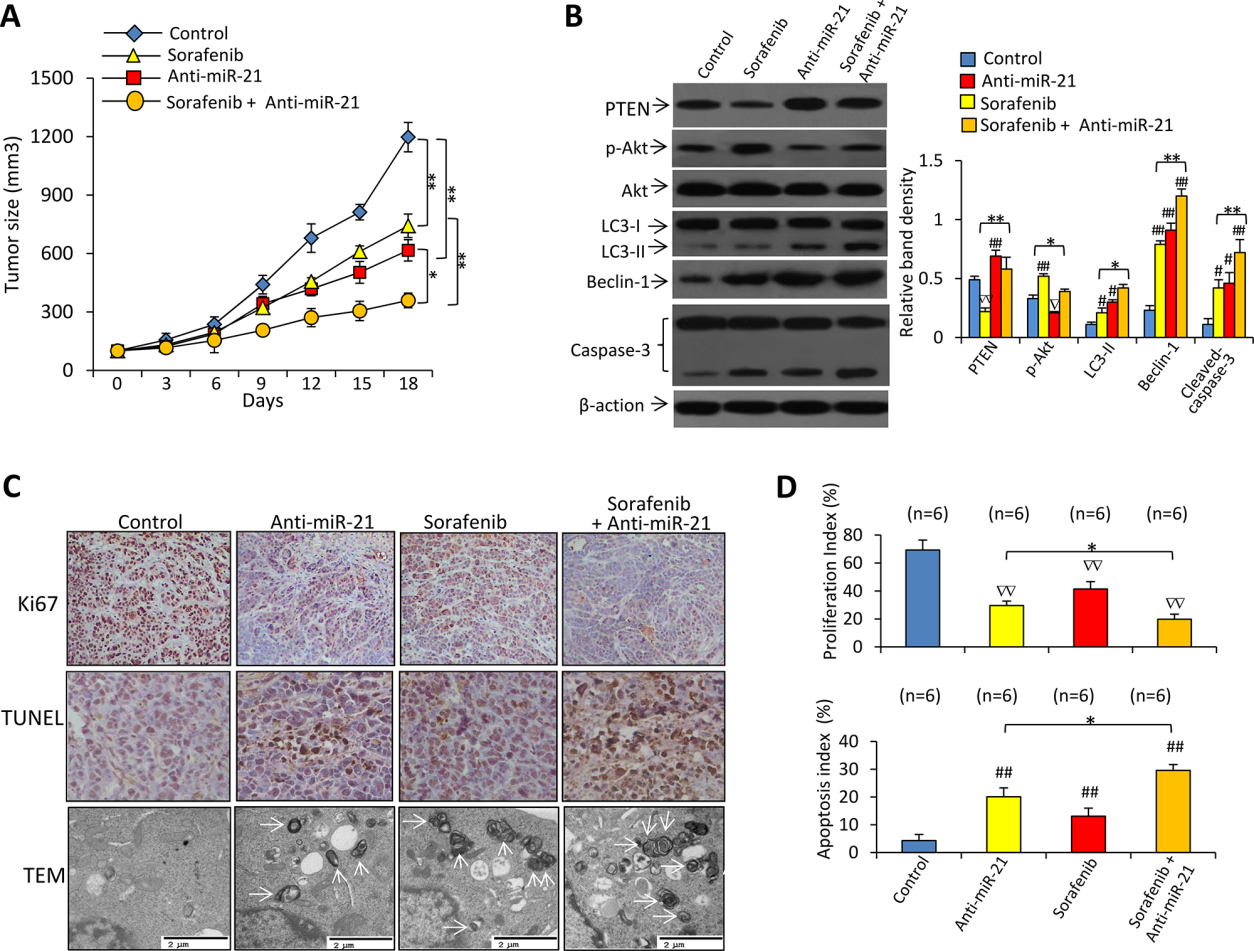
Inhibition of miR-21 enhances the efficacy of sorafenib to suppress sorafenib-resistant tumors *in vivo* **A.** Subcutaneous tumors were established in mice, which received different treatments for 18 days as described in Materials and Methods. **B.** Immunoblots of lysates of tumors harvested at the end of experiments. The density of each band was measured and normalized to respective β-actin. **C.** Sections of tumors were stained with an anti-Ki67 Ab (upper panel, magnification ×100) or TUNEL (middle panel, magnification ×200), or viewed under transmission electron microscopy (TEM). Arrows point to autophagosomes. **D.** Proliferation index and apoptosis index were quantified. “n” indicates the number of samples examined. “*” (*P* < 0.05) and “**” (*P* < 0.001) indicate a significant difference. “#” (*P* < 0.05) and “##” (*P* < 0.001) indicate a significant increase; while “∇” (*P* < 0.05) and “∇∇” (*P* < 0.001), a significant reduction, versus controls.

In animal studies, oral administration of sorafenib and intratumoral injection of anti-miR-21 oligonucleotides significantly reduced the size of Huh7-SR tumors by 29.6% and 51.5%, respectively at day 18. The combinational therapy resulted in a further reduction by 74.5%, compared with control tumors (Figure 7A).

Immunoblotting analysis of tumor lysates demonstrated that sorafenib treatment resulted in downregulation of PTEN, increased the activation of Akt and caspase-3, and upregulated LC3-II and Beclin-1 expression (Figure 7B). Treatment of anti-miR-21 led to upregulation of PTEN, LC3-II, Beclin-1 and cleaved caspase-3, and reduced expression of p-Akt (Figure 7B). There were fewer Ki-67 positive cells in tumors treated with anti-miR-21 or sorafenib, compared with control tumors; and the combinational therapy resulted in even fewer Ki-67 positive cells (Figure 7C, 7D). Tumors treated with anti-miR-21 or sorafenib had a greater number of TUNEL-positive cell than control tumors, and the combinational therapy resulted in even more TUNEL-positive cells (Figure 7C, 7D). Electron microscopy also revealed abundant autophagosomes in cells from tumors treated by miR-21 inhibitor plus sorafenib, but they were rare in vehicle-treated tumors (Figure 7C).

## DISCUSSION

The discovery of sorafenib has opened a narrow window of hope for combating HCC, but this promising treatment has demonstrated low survival benefits, and some HCC patients initially respond to sorafenib but eventually the disease progresses [3]. Unfortunately, there are no alternative effective systemic agents against HCC currently available [39, 40]. Therefore, it is essential to investigate underlying mechanisms for the acquired resistance to sorafenib and seek potential strategies to increase its efficacy against HCC. In this report, we show that up-regulation of miR-21 is induced in the acquired resistance to sorafenib in HCC cells. This increase is likely to contribute to the resistance by inhibiting sorafenib-induced autophagy through downregulating PTEN and sequential activation of the Akt pathway. Transfection of miR-21 mimics into parental HCC cells rendered cells insensitive to sorafenib-induced growth inhibition and apoptosis by inhibiting cell autophagy. Antagonism of miR-21 by specific oligonucleotides re-sensitized sorafenib-resistant cells to sorafenib by promoting autophagy, and increases the efficacy of sorafenib in treating sorafenib-resistant HCC tumors established in mice.

Although it is not a direct target of sorafenib, the PI3K/Akt pathway plays a critical role in the mechanism of sorafenib resistance as it crosstalks with the major sorafenib-targeted MAPK/ERK pathway [5]. It has been previously demonstrated that sorafenib activates Akt and blockade of the PI3K/Akt pathway increases the efficacy of sorafenib [9–11]. Here we have again shown that sorafenib induces the activation of Akt in HCC cells, in accordance with our previous report [13]. Akt upregulates mTOR expression and the phosphorylation of glycogen synthase kinase (GSK)3β, which regulate apoptotic proteins S6K and 4EBP1 [12, 41]. The mTOR also controls autophagy by regulating autophagic proteins LC3 and Beclin-1 [37].

Sustained drug exposure can induce an imbalance in apoptotic pathways, leading to resistance to apoptosis [42], and enabling an autophagy switch from a protective to a death-promoting role [13, 28, 43, 44]. Beclin-1 binds to other components to form a core complex to allow autophagosome nucleation [45], and interacts with apoptotic molecules [28]. It has been reported that paclitaxel resistance is associated with a switch from apoptotic to autophagic cell death in breast cancer cells [44]. Crosstalks between autophagic and apoptotic pathways offer opportunities for therapeutic intervention [28]. Therefore, inhibition of mTOR by RAP promoted cell autophagic death and thus augmented the effects of sorafenib against sorafenib-resistant HCC cells as in this study and supported by a previous report [46].

Altered expression and dysfunction of miRNAs have been well documented in carcinogenesis and progression of human malignancies by regulating the expression of oncogenes and suppressor oncogenes. MiR-21 is one of the few miRNAs that are expressed in various types of malignances including HCC [15, 16, 17]. The recently proposed “onco-miR addition” concept has further strengthened the central role of miR-21 in carcinogenesis [47]. MiR-21 has also been identified as the one molecular species associated with drug resistance [22]. Overexpressed miR-21 contributes to the resistance of HCC cells to interferon-α/5-fluorouracil [25], the acquired resistance of EGFR-TKI [23] and gefitinib resistance [24] in non-small cell lung cancer cells [23], and the acquired resistance to trastuzumab in breast cancer [20]. Blockage of miR-21 augments the efficacy of anti-EGFR therapy against glioblastoma [21]. In accord, we reported herein that overexpression of miR-21 is induced in the acquired resistance to sorafenib and inhibition of miR-21 enhances the efficacy of sorafenib in the treatment of sorafenib-resistant HCC cells.

Among the miR-21 targets, PTEN is a well characterized tumor-suppressing phosphatase that inhibits Akt activation. By downregulating PTEN, miR-21 promotes the growth of HCC through the Akt pathway [15, 17]. Our results demonstrate that PTEN reduction due to elevated miR-21 is responsible for sorafenib resistance in HCC cells. By rescuing PTEN expression in sorafenib-resistant cells, anti-miR-21 oligonucleotides led to Akt inhibition. Accordingly, antagonism of miR-21 inactivated Akt through PTEN upregulation in HCC cells [17]. On the other hand, transfection of miR-21 mimics inhibited the expression of PTEN, leading to activation of AKT, and promoting HCC cell growth, in accordance with a previous report [19]. Although it has not been investigated here, the regulation of Bcl-2 by miR-21 may also participate in sorafenib resistance, as ani-miR-21 was shown to increase autophagy and chemosensitivity of leukemia cells by upregulating Bcl-2 expression [34].

In addition, the present study has also demonstrated that miR-21-inhibited autophagy contributes to the resistance to sorafenib, possibly through regulating the PTEN/Akt pathway [32]. Transfection of miR-21 mimics inhibited autophagy, while anti-miR-21 oligonucleotides promoted autophagy of HCC cells. Inhibition of miR-21 enhances the efficacy of sorafenib against sorafenib-resistant HCC cells by promoting cell autophagy. Therfore, blockade of miR-21 increased the sensitivity of radiotherapy against malignant glioma cells [33], and chemosensitivity of leukemia cells [34], through enhancing autophagy.

In summary, the results of this study demonstrate a significant association between miR-21 expression and the acquired resistance of sorafenib in HCC. The miR-21-mediated sorafenib resistance is through its inhibitory effects on autophagy by regulating the PTEN/Akt pathway. Our findings suggest that miR-21 could be a potentially therapeutic target for reversing the sorafenib resistance in treating HCC.

## MATERIALS AND METHODS

### Cell culture, antibodies and reagents

Human HCC HepG2 cells were obtained from the American Type Culture Collection (ATCC, Manassas, VA, USA), and Huh7 cells from Chinese Academy of Sciences Cell Bank (Shanghai, China). Cells were cultured at 37°C in Dulbecco's modified Eagle's medium (DMEM) (Gibco BRL, Grand Island, NY, USA) supplemented with 10% fetal bovine serum. The antibodies (Abs) against Akt, p-Akt (Ser473), GSK3β, phosphorylated GSK3β (p-GSK3β) (Ser9), mTOR, phosphorylated mTOR (p-mTOR) (Ser2448), S6K, phosphorylated S6K (p-S6K) (Thr389), 4EBP1, phosphorylated 4EBP1 (p-4EBP1) (Ser65), LC3, Beclin-1 and p62 were purchased from Cell Signaling Technology (Danvers, USA). The Abs against PTEN, caspase-3 and -9 and β-actin were from Santa Cruz Biotechnology (CA, USA). The anti-Ki67 Ab was from Abcam (Cambridge, MA, USA). Sorafenib was from Jinan Trio Pharmatech Co., Ltd. (Jinan, China). RAP, 3-MA, E-64D, pepstatin A, acridine orange and MD) were from Sigma-Aldrich (Shanghai, China). Sorafenib and RAP were dissolved in dimethyl sulfoxide (DMSO) to make a stock solution of 100 mM and 1 mM for *in vitro* assays, respectively. 3-MA was dissolved in PBS at a concentration of 200 mM by heating to 60–70°C immediately before use.

### Establishment of sorafenib-resistant cells

The half maximal inhibitory concentration (IC_50_) of HCC cells to sorafenib was initially determined by incubating cells with different concentrations of sorafenib in 96-well plates, and cell viability was measured 3 days later as described below. The cells were cultured in 6-well plates at 1 × 10^4^ cells/well and incubated with sorafenib at a concentration just below their respective IC_50_. The concentration of sorafenib was slowly increased by 0.25 μM per week. After 6 months, two sorafenib-resistant cell lines were obtained, termed HepG2-SR and Huh7-SR, respectively, and were continuously maintained by culturing them in the presence of sorafenib.

### MiRNA microarray and real-time PCR miRNA quantification

Total RNA was extracted from cells using Trizol reagent (Invitrogen). After reverse transcription with a TaqMan MicroRNA Reverse Transcription kit (Applied Biosystems), PCR array assays were performed by using TagMan Human MicroRNA Array A + B cards set v3.0 (Applied Biosystems). The 754 human mature miRNAs and 3 endogenous controls were reversely transcribed using Megaplex PreAmp primers. The reverse transcription products were subsequently loaded onto the TaqMan array to do real-time PCR amplification by using MX3000P Real-time PCR system (Stratagen, USA). The expression of miRNAs was measured by the ΔΔCt methods. The ΔCt was calculated by subtracting the Ct of U6 RNA from the Ct of each miRNA of interest. The ΔΔCt was calculated by subtracting the ΔCt of the control sample (parental Huh7 cells) from the ΔCt of each sample (Huh7-SR cells). Fold change was generated by using the equation 2^−ΔΔCt^.

To validate the expression levels of miRNAs measured by microarray, several selected miRNAs and U6 (an inner control) were further examined by real-time PCR by using a TaqMan MiRNA Reverse Transcription Kit (Applied Biosystems), individual TaqMan MiRNA assay, and MX3000P Real-time PCR system.

### Luciferase reporter assay

To evaluate the function of miR-21, the 3′UTR of PTEN with a miR-21 targeting sequence was cloned into a pMIR-REPORT luciferase reporter vector (Ambion). The assay was conducted as described previously [20]. Briefly, the reporter vector plasmid was transfected into cells using Lipofectamine 2000. To correct transfection efficiency, a luciferase reporter vector without the miR-21 target was transfected in parallel. Luciferase activities in cells were measured by using a luciferase assay kit (Promega, Madison, WI), and a miR-21 function was expressed as percentage of the luciferase activity of the reporter vector with miR-21 target sequence over one without the miR-21 sequence.

### Transfection of oligonucleotides

MiR-21 mimics (5′-AACAUCAGUCUGAUAAGC UAUU-3′), anti-miR-21 (5′-UCAACAUCAGUCUGAUA AGCUA-3′) and the negative control oligonucleotides (5′-CAGUACUUUUGUGUAGUACAA-3′) were purchased from GenePharma Co., Ltd., Shanghai, China). Cells were grown to 60–70% confluence, and incubated with RNAs at a final concentration of 0.1 μM by using Lipofectamine^TM^ 2000 (Invitrogen, Beijing, China) in a serum-free medium for 48 h and then subjected to assays.

### Autophagy assays

Cells were incubated with acridine orange (5 μM) at 37°C for 15 min, washed with cold PBS, and examined by fluorescent microscopy. AVOs appeared as orange/red fluorescent cytoplasmic vesicles, while nuclei were stained green. Acridine orange-stained cells were further trypsinized and analyzed on a FACScalibur flow cytometer (BD. Biosciences, San Jose, California, USA). The degree of autophagic lysosome was expressed as fold change of acridine orange fluorescence intensity (FL3) of red in treated cells versus control cells. Autophagic vacuoles were also detected with MDC (Sigma-Aldrich) staining. Briefly, cells were washed with PBS and then incubated with 0.05 mM MDC in PBS at 37°C for 45 min. After incubation, the cells were washed four times with PBS and immediately analyzed by flow cytometry, and viewed by fluorescence microscopy. Tumor tissues were fixed in 2.5% glutaraldehyde solution for 1 h, washed twice with PBS, followed by further fixation with 1% Osmic acid for 1 h, dehydrated with a graded series of ethanol, embedded, and sectioned. Sections were stained with uranium acetate and lead citrate, and observed under a transmission electron microscope (JEN-M1220, Toshiba, Japan) [13].

### Animal experiments

Six to 8-week-old male nude BALB/c mice (H-2b) were obtained from the Animal Research Center, The First Affiliated Hospital of Harbin Medical University, China. This study had been approved (permit SYXK20020009) by the Animal Ethics Committee of Harbin Medical University, in compliance with the Experimental Animal Regulations by the National Science and Technology Commission, China. The experimental protocol has been described previously [13, 35, 36]. Briefly, Huh7-SR cells (5 × 10^6^) were inoculated subcutaneously into the back of mice, which received oral administration of 15 mg/kg sorafenib every three days. The use of lower dose of sorafenib was to maintain the sorafenib-resistant ability of Huh7-SR cells, which were kept in the presence of sorafenib in culture. Thirty days later (when the tumors reached ∼100 mm^3^), mice were assigned to four treatment groups (*n* = 6 per group), namely control, sorafenib, anti-miR-21, and sorafenib + anti-miR-21 groups. Sorafenib was suspended in an oral vehicle containing Cremophor (Sigma-Aldrich), 95% ethanol and water in a ratio of 1:1:6 [13, 35], and administrated orally at a dose of 30 mg/kg by gavage daily. Anti-miR-21 or negative control oligonucleotides were mixed with Lipofectamine2000 (5 pmol/μl of oligonucleotides solution), and injected intratumorally at multiple sites every 3 days. Mice in the control group received intratumoral control oligonucleotides and oral vehicle; mice in sorafenib group, oral sorafenib and intratumoral control oligonucleotides; mice in the anti-miR-21 group, oral vehicle and intratumoral anti-miR-21 oligonucleotides; mice in the sorafenib + anti-miR-21 group, oral sorafenib and intratumoral anti-miR-21 oligonucleotides. Tumor were measured every 3 days and harvested 18 days following treatment commencement.

### Cell proliferation analysis

The cells were seeded into a 96-well plate (3 × 10^3^/well) in triplicate, and cultured overnight. The culture medium was replaced with fresh FCS-free media containing vehicle or testing reagents at various concentrations for 24, 48 or 72 h. Cell viability was measured with a Cell Counting Kit-8 (CCK-8) kit (Dojindo Molecular Technologies, Gaithersburg, MD, USA). Untreated cells served as controls. Cell viability (%) was calculated according to the formula: experimental OD value/control OD value × 100%.

### *In vitro* apoptosis assay

Cells (1 × 10^5^) were suspended in 100μl binding buffer, 5μl of Annexin V and 5μl of propidium iodide (PI) were added, and incubated for 15 min at room temperature in dark, according to the manufacturer's instruction (BD Biosciences, San Jose, CA). Then the cells were subjected to flow cytometry to measure the apoptosis rate (%) with a Beckman Coulter Epics Altra II cytometer (Beckman Coulter, California, USA). The experiments were repeated thrice.

### Immunoblotting analysis

Cells or tumor tissues were homogenized in protein lysate buffer (50 mmol/L Tris pH 7.4, 100 μmol/L EDTA, 0.25mol/L sucrose, 1%SDS, 1% NP40, 1 μg/ml leupeptin, 1 μg/ml pepstatin A and 100 μmol/L phenyl methyl sulfonyl flouride) and debris was removed by centrifugation at 10,000 × g for 10 min at 4°C. Protein concentrations were determined (Bio-Rad, Richmond, CA, USA). Lysates were resolved on sodium dodecyl sulfate-polyacrylamide (SDS-PAGE) gels, electrophoretically transferred to polyvinylidene difluoride (PVDF) membranes. The membranes were blocked in TBST (137 mM NaCl, 20 mM Tris HCl [pH 7.6], and 0.1% [v/v] Tween 20) containing 5% (w/v) nonfat dry milk at 37°C for 2 h, and then incubated overnight with primary Abs, and subsequently with alkaline phosphatase-conjugated secondary Abs for 2 h at room temperature in the dark. They were developed with 5-bromo-4-chloro-3-indolyl phosphate (BCIP)/ nitro blue tetrazolium (NBT) (Tiangen Biotech Co. Ltd., Beijing, China). The density of each band was measured using a densitometric analysis program (FR200, Shanghai, China), and normalized to that of β-actin from the same cells. In preliminary experiments, serial dilutions of lysates (containing 2.5, 5, 10, 20, 40 or 80 μg protein) were immunoblotted; band intensities were measured and plotted against protein amounts to generate a standard curve, and the amount of protein for each blot was determined.

### Real-time RT-PCR for detecting PTEN mRNA

The methods have been described in details previously [37, 38]. Briefly, total RNA was extracted from cells, and cDNA was synthesized. The reaction mixtures for real-time RT-PCR were prepared with the primers for PTEN mRNA (Forward: 5′-CAAGATGATGTTTGAAACTATTCCAATG-3′ and Reverse: 5′-CCTTTAGCTGGCAGACCACAA-3′) and an internal control GAPDH mRNA (Forward: 5′-CACCCATGGCAAATTCCATGGCA-3′ and Reverse: 5′-TCTAGACGGCAGGTCAGGTCCACC-3′). The PCR products were analyzed by MX3000P Real-time PCR systems (Stratagen, USA). Experiments were performed in triplicate, and data were calculated by ΔΔCt methods.

### *In situ* Ki-67 proliferation index

Formalin fixed tumor specimens were transferred to 70% ethanol, and subsequently paraffin-embedded and sectioned. Tumor sections were rinsed with PBS, blocked with 3% BSA for 2 h, and incubated with an anti-Ki-67 Ab at 4°C overnight. They were subsequently incubated for 30 min with the appropriate secondary Ab using the Ultra-Sensitive TMS-P kit (Zhongshan Co., Beijing, China), and immunoreactivity developed with Sigma FAST DAB (3, 3′-diaminobenzidine tetrahydrochloride) and CoCl_2_ enhancer tablets (Sigma-Aldrich, Shanghai, China). Sections were counterstained with hematoxylin, mounted, and examined by microscopy. The Ki-67 positive cells were counted in 10 randomly selected × 400 high-power fields under microscopy. The Ki-67 proliferation index was calculated according to the following formula: the number of Ki-67 positive cells/ the total cell count × 100%.

### *In situ* detection of apoptotic cells

The above tumor sections were stained with the TUNEL (Terminal deoxynucleotidyl transferase dUTP nick end labelling) (Roche, Shanghai, China). The TUNEL positive cells were counted in 20 randomly selected × 200 high-power fields under microscopy. The apoptosis index was calculated according to the following formula: the number of apoptotic cells × /total number of nucleated cells × 100%.

### Statistical analysis

All the data are presented as the mean ± standard deviation. Comparisons of the paired data were carried out using *t*-test, and comparisons among multiple groups were carried out using one-way analysis of variance followed by Dunnet's *t*-test with the SPSS 13.0 software package. *P* < 0.05 indicates a statistical significance.

## SUPPLEMENTARY FIGURES AND TABLE



## References

[1] Jemal A, Bray F, Center MM, Ferlay J, Ward E, Forman D (2011). Global cancer statistics. CA Cancer J Clin.

[2] Zhu AX (2010). Systemic treatment of hepatocellular carcinoma: dawn of a new era?. Ann Surg Oncol.

[3] Llovet JM, Ricci S, Mazzaferro V, Hilgard P, Gane E, Blanc JF, de Oliveira AC, Santoro A, Raoul JL, Forner A, Schwartz M, Porta C, Zeuzem S (2008). Sorafenib in advanced hepatocellular carcinoma. N Engl J Med.

[4] Cervello M, McCubrey JA, Cusimano A, Lampiasi N, Azzolina A, Montalto G (2012). Targeted therapy for hepatocellular carcinoma: novel agents on the horizon. Oncotarget.

[5] Aksamitiene E, Kiyatkin A, Kholodenko BN (2012). Cross-talk between mitogenic Ras/MAPK and survival PI3K/Akt pathways: a fine balance. Biochem Soc Trans.

[6] Ohta K, Hoshino H, Wang J, Ono S, Iida Y, Hata K, Huang SK, Colquhoun S, Hoon DS (2015). MicroRNA-93 activates c-Met/PI3K/Akt pathway activity in hepatocellular carcinoma by directly inhibiting PTEN and CDKN1A. Oncotarget.

[7] Zhou Q, Lui VW, Yeo W (2011). Targeting the PI3K/Akt/mTOR pathway in hepatocellular carcinoma. Future Oncol.

[8] Chen KF, Chen HL, Tai WT, Feng WC, Hsu CH, Chen PJ, Chen AL (2011). Activation of phosphatidylinositol 3-kinase/Akt signaling pathway mediates acquired resistance to sorafenib in hepatocellular carcinoma cells. J Pharmacol Exp Ther.

[9] Gedaly R, Angulo P, Hundley J, Daily MF, Chen C, Koch A, Evers BM (2010). PI-103 and sorafenib inhibit hepatocellular carcinoma cell proliferation by blocking Ras/Raf/MAPK and PI3K/AKT/mTOR pathways. Anticancer Res.

[10] Huynh H, Ngo VC, Koong HN, Poon D, Choo SP, Thng CH, Chow P, Ong HS, Chung A, Soo KC (2009). Sorafenib and rapamycin induce growth suppression in mouse models of hepatocellular carcinoma. J Cell Mol Med.

[11] Piguet AC, Saar B, Hlushchuk R, St-Pierre MV, McSheehy PM, Radojevic V, Afthinos M, Terracciano L, Djonov V, Dufour JF (2011). Everolimus augments the effects of sorafenib in a syngeneic orthotopic model of hepatocellular carcinoma. Mol Cancer Ther.

[12] Serova M, de Gramont A, Tijeras-Raballand A, Dos Santos C, Riveiro ME, Slimane K, Faivre S, Raymond E (2013). Benchmarking effects of mTOR, PI3K, and dual PI3K/mTOR inhibitors in hepatocellular and renal cell carcinoma models developing resistance to sunitinib and sorafenib. Cancer Chemother Pharmacol.

[13] Zhai B, Hu F, Jiang X, Xu J, Zhao D, Liu B, Pan S, Dong X, Tan G, Wei Z, Qiao H, Jiang H, Sun X (2014). Inhibition of Akt reverses the acquired resistance to sorafenib by inducing autophagic cell death in hepatocellular carcinoma. Cancer Mol Ther.

[14] Di Leva G, Garofalo M, Croce CM (2014). MicroRNAs in cancer. Annu Rev Pathol.

[15] Meng F, Henson R, Wehbe-Janek H, Ghoshal K, Jacob ST, Patel T (2007). MicroRNA-21 regulates expression of the PTEN tumor suppressor gene in human hepatocellular cancer. Gastroenterology.

[16] Connolly E, Melegari M, Landgraf P, Tchaikovskaya T, Tennant BC, Slagle BL, Rogler LE, Zavolan M, Tuschl T, Rogler CE (2008). Elevated expression of the miR-17–92 polycistron and miR-21 in hepadnavirus-associated hepatocellular carcinoma contributes to the malignant phenotype. Am J Pathol.

[17] Bao L, Yan Y, Xu C, Ji W, Shen S, Xu G, Zeng Y, Sun B, Qian H, Chen L, Wu M, Su C, Chen J (2013). MicroRNA-21 suppresses PTEN and hSulf-1 expression and promotes hepatocellular carcinoma progression through AKT/ERK pathways. Cancer Lett.

[18] Si ML, Zhu S, Wu H, Lu Z, Wu F, Mo YY (2007). miR-21-mediated tumor growth. Oncogene.

[19] Liu LZ, Li C, Chen Q, Jing Y, Carpenter R, Jiang Y, Kung HF, Lai L, Jiang BH (2011). MiR-21 induced angiogenesis through AKT and ERK activation and HIF-1α expression. PLoS One.

[20] Gong C, Yao Y, Wang Y, Liu B, Wu W, Chen J, Su F, Yao H, Song E (2011). Up-regulation of miR-21 mediates resistance to trastuzumab therapy for breast cancer. J Biol Chem.

[21] Zhang KL, Han L, Chen LY, Shi ZD, Yang M, Ren Y, Chen LC, Zhang JX, Pu PY, Kang CS (2014). Blockage of a miR-21/EGFR regulatory feedback loop augments anti-EGFR therapy in glioblastomas. Cancer Lett.

[22] Hong L, Han Y, Zhang Y, Zhang H, Zhao Q, Wu K, Fan D (2013). MicroRNA-21: a therapeutic target for reversing drug resistance in cancer. Expert Opin Ther Targets.

[23] Li B, Ren S, Li X, Wang Y, Garfield D, Zhou S, Chen X, Su C, Chen M, Kuang P, Gao G, He Y, Fan L (2014). MiR-21 overexpression is associated with acquired resistance of EGFR-TKI in non-small cell lung cancer. Lung Cancer.

[24] Shen H, Zhu F, Liu J, Xu T, Pei D, Wang R, Qian Y, Li Q, Wang L, Shi Z, Zheng J, Chen Q, Jiang B (2014). Alteration in Mir-21/PTEN expression modulates gefitinib resistance in non-small cell lung cancer. PLoS One.

[25] Tomimaru Y, Eguchi H, Nagano H, Wada H, Tomokuni A, Kobayashi S, Marubashi S, Takeda Y, Tanemura M, Umeshita K, Doki Y, Mori M (2010). MicroRNA-21 induces resistance to the anti-tumour effect of interferon-α/5-fluorouracil in hepato-cellular carcinoma cells. Br J Cancer.

[26] David R (2012). Metabolism: Keeping fit with autophagy. Nat Rev Mol Cell Biol.

[27] Codogno P, Mehrpour M, Proikas-Cezanne T (2012). Canonical and non-canonical autophagy: variations on a common theme of self-eating?. Nat Rev Mol Cell Biol.

[28] Su M, Mei Y, Sinha S (2013). Role of the Crosstalk between Autophagy and Apoptosis in Cancer. J Oncol.

[29] Tai WT, Shiau CW, Chen HL, Liu CY, Lin CS, Cheng AL, Chen PJ, Chen KF (2013). Mcl-1-dependent activation of Beclin 1 mediates autophagic cell death induced by sorafenib and SC-59 in hepatocellular carcinoma cells. Cell Death Dis.

[30] Funderburk SF, Wang QJ, Yue Z (2010). The Beclin 1-VPS34 complex—at the crossroads of autophagy and beyond. Trends Cell Biol.

[31] Sini P, James D, Chresta C, Guichard S (2010). Simultaneous inhibition of mTORC1 and mTORC2 by mTOR kinase inhibitor AZD8055 induces autophagy and cell death in cancer cells. Autophagy.

[32] Ueno T, Sato W, Horie Y, Komatsu M, Tanida I, Yoshida M, Ohshima S, Mak TW, Watanabe S, Kominami E (2008). Loss of Pten, a tumor suppressor, causes the strong inhibition of autophagy without affecting LC3 lipidation. Autophagy.

[33] Gwak HS, Kim TH, Jo GH, Kim YJ, Kwak HJ, Kim JH, Yin J, Yoo H, Lee SH, Park JB (2012). Silencing of microRNA-21 confers radio-sensitivity through inhibition of the PI3K/AKT pathway and enhancing autophagy in malignant glioma cell lines. PLoS One.

[34] Seca H, Lima RT, Lopes-Rodrigues V, Guimaraes JE, Almeida GM, Vasconcelos MH (2013). Targeting miR-21 induces autophagy and chemosensitivity of leukemia cells. Curr Drug Targets.

[35] Ma L, Li G, Zhu H, Dong X, Zhao D, Jiang X, Li J, Qiao H, Ni S, Sun X (2014). 2-Methoxyestradiol synergizes with sorafenib to suppress hepatocellular carcinoma by simultaneously dysregulating hypoxia-inducible factor-1 and -2. Cancer Lett.

[36] Zhao D, Zhai B, He C, Tan G, Jiang X, Pan S, Dong X, Wei Z, Ma L, Qiao H, Jiang H, Sun X (2014). Upregulation of HIF-2α induced by sorafenib contributes to the resistance by activating the TGF-α/EGFR pathway in hepatocellular carcinoma cells. Cell Sig.

[37] Sini P, James D, Chresta C, Guichard S (2010). Simultaneous inhibition of mTORC1 and mTORC2 by mTOR kinase inhibitor AZD8055 induces autophagy and cell death in cancer cells. Autophagy.

[38] Jung CH, Ro SH, Cao J, Otto NM, Kim DH (2010). mTOR regulation of autophagy. FEBS Lett.

[39] Villanueva A, Llovet JM (2012). Second-line therapies in hepatocellular carcinoma: emergence of resistance to sorafenib. Clin Cancer Res.

[40] Santoro A, Rimassa L, Borbath I, Daniele B, Salvagni S, Van Laethem JL, Van Vlierberghe H, Trojan J, Kolligs FT, Weiss A, Miles S, Gasbarrini A, Lencioni M (2013). Tivantinib for second-line treatment of advanced hepatocellular carcinoma: a randomised, placebo-controlled phase 2 study. Lancet Oncol.

[41] Hennessy BT, Smith DL, Ram PT, Lu Y, Mills GB (2005). Exploiting the PI3K/AKT pathway for cancer drug discovery. Nat Rev Drug Discov.

[42] Lackner MR, Wilson TR, Settleman J (2012). Mechanisms of acquired resistance to targeted cancer therapies. Future Oncol.

[43] Shimizu S, Takehara T, Hikita H, Kodama T, Tsunematsu H, Miyagi T, Hosui A, Ishida H, Tatsumi T, Kanto T, Hiramatsu N, Fujita N, Yoshimori T, Hayashi N (2012). Inhibition of autophagy potentiates the antitumor effect of the multikinase inhibitor sorafenib in hepatocellular carcinoma. Int J Cancer.

[44] Ajabnoor GM, Crook T, Coley HM (2012). Paclitaxel resistance is associated with switch from apoptotic to autophagic cell death in MCF-7 breast cancer cells. Cell Death Dis.

[45] Pattingre S, Tassa A, Qu X, Garuti R, Liang XH, Mizushima N, Packer M, Schneider MD, Levine B (2005). Bcl-2 antiapoptotic proteins inhibit Beclin 1-dependent autophagy. Cell.

[46] Serova M, de Gramont A, Tijeras-Raballand A, Dos Santos C, Riveiro ME, Slimane K, Faivre S, Raymond E (2013). Benchmarking effects of mTOR, PI3K, and dual PI3K/mTOR inhibitors in hepatocellular and renal cell carcinoma models developing resistance to sunitinib and sorafenib. Cancer Chemother Pharmacol.

[47] Medina PP, Nolde M, Slack FJ (2010). OncomiR addiction in an *in vivo* model of microRNA-21-induced pre-B-cell lymphoma. Nature.

