# Supplementary material for: MiR-21 mediates sorafenib resistance of hepatocellular carcinoma cells by inhibiting autophagy via the PTEN/Akt pathway
Source: Oncotarget. 2015 Jul 30;6(30):28867–81. doi: 10.18632/oncotarget.4814 (PMC4745697; doi:10.18632/oncotarget.4814)
Supplement: Supplementary file 1 [file oncotarget-06-28867-s001.pdf]

SUPPLEMENTARY FIGURES AND TABLE

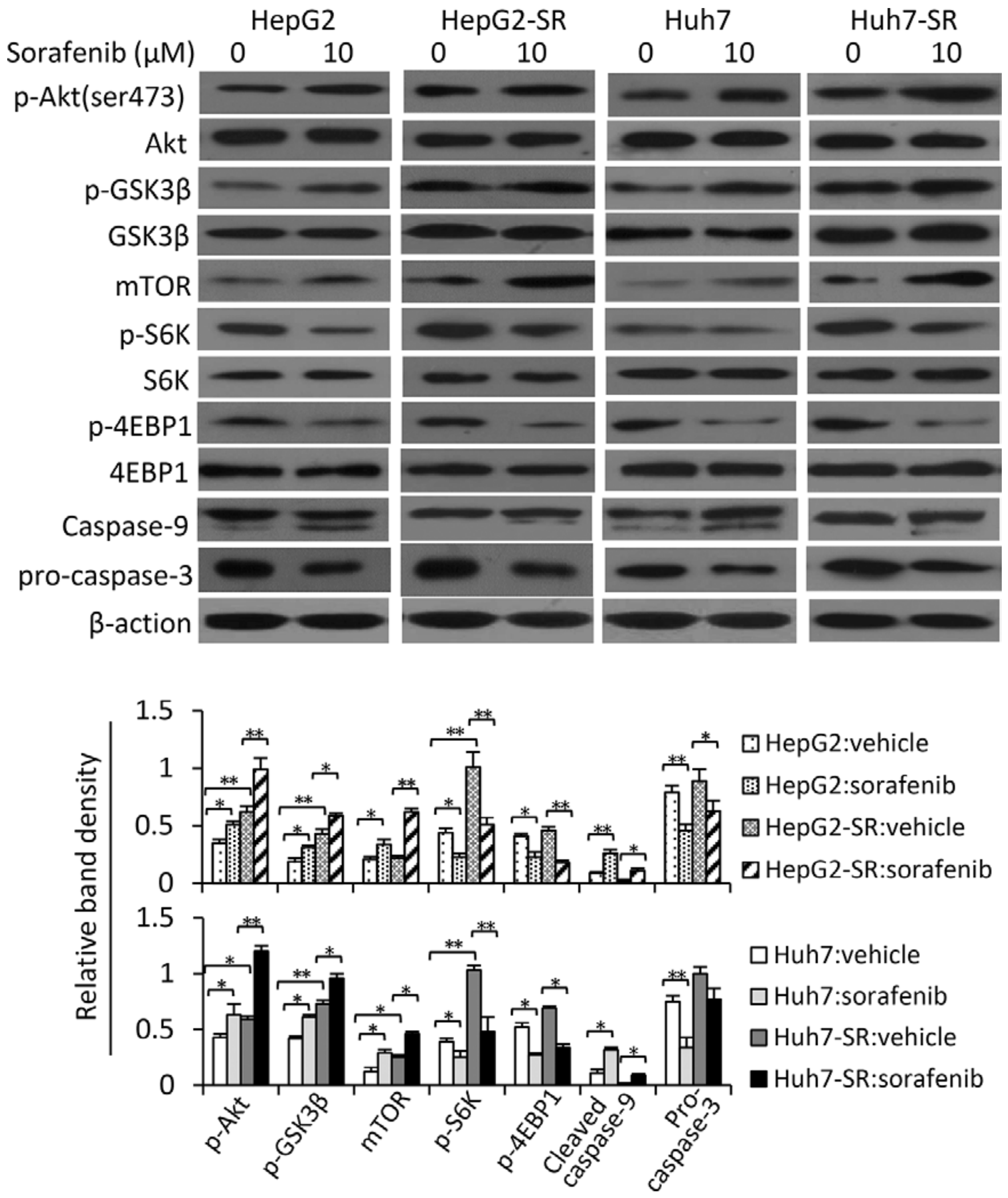

Supplementary Figure S1: Sorafenib activates the Akt pathway in HCC cells. HepG2, HepG2-SR, Huh7 and Huh7-SR cells were incubated with sorafenib (0, 10 μM) for 48 h, harvested and immunoblotted. The density of each band was measured and normalized to respective β-actin. *($P < 0.05$) and **($P < 0.001$) indicate a significant difference.

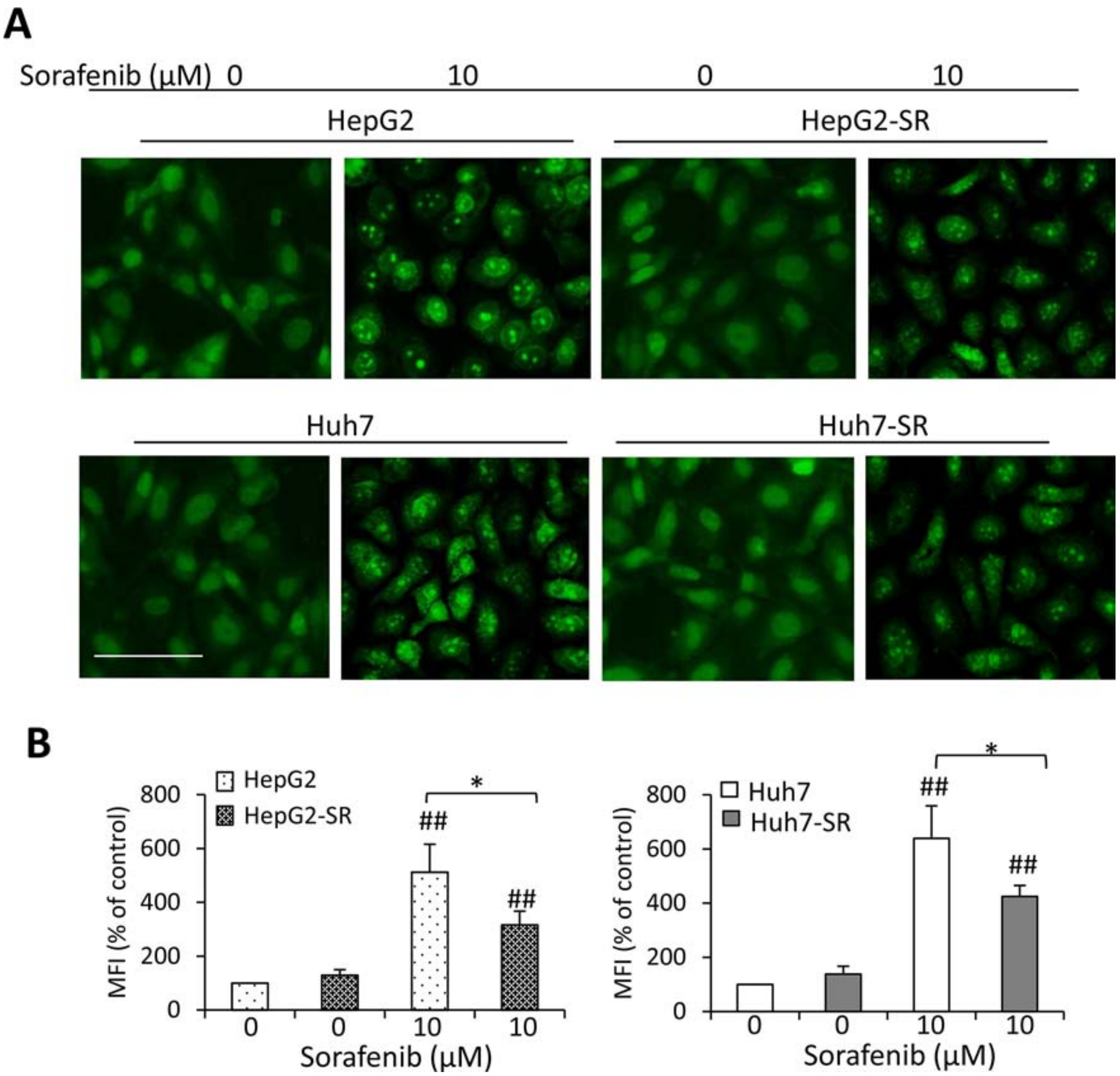

Supplementary Figure S2: Autophagy assay by monodansycadaverine (MDC) staining. HepG2, HepG2-SR, Huh7 or Huh7-SR cells were incubated sorafenib (0, 10 μM) for 48 h, and then stained by MDC. **A.** Representative images from the above cells stained by MDC. Original magnification: 400x, scale bar =500 μm. **B.** The mean fluorescence intensity (MFI) (% of control) was measured by flow cytometry. Untreated parental cells served as controls. *(*P* < 0.05) indicates a significant difference. ##(*P* < 0.001) indicates a significant increase from respective controls.

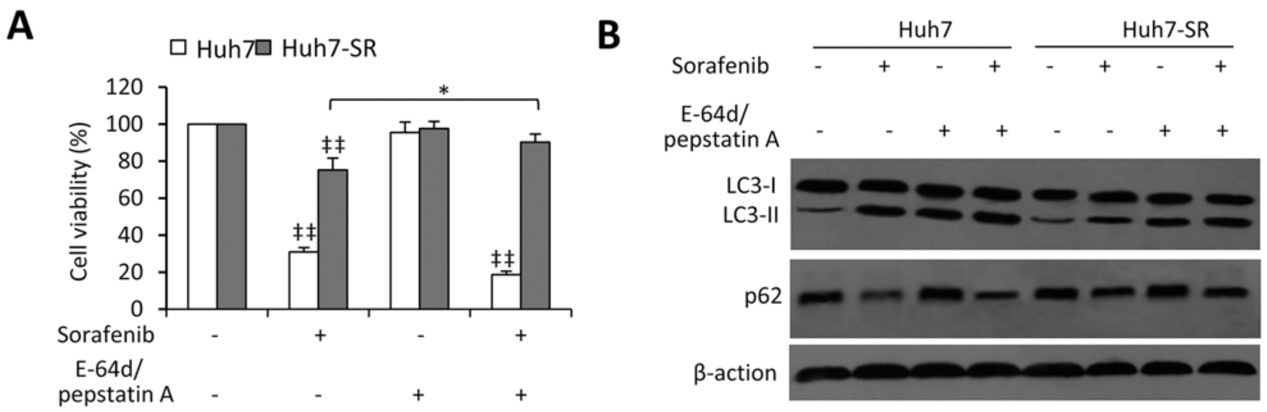

Supplementary Figure S3: The effects of inhibition of late-stage autophagy on sorafenib-induced reduction of cell viability in HCC cells. Huh7 or Huh7-SR cells were incubated for 48 h in the presence or absence of sorafenib **(**10 μM), a mixture of E-64d (10 μg/ml) and pepstatin A (10 μg/ml), or the combination. **A.** Cell viability (%) was compared the corresponding untreated cells. *($P < 0.05$) indicates a significant difference."‡‡" ($P < 0.001$) indicates a significant reduction versus respective untreated cells. **B.** Cell lysates were immunoblotted to detect expression of LC3 and p62.

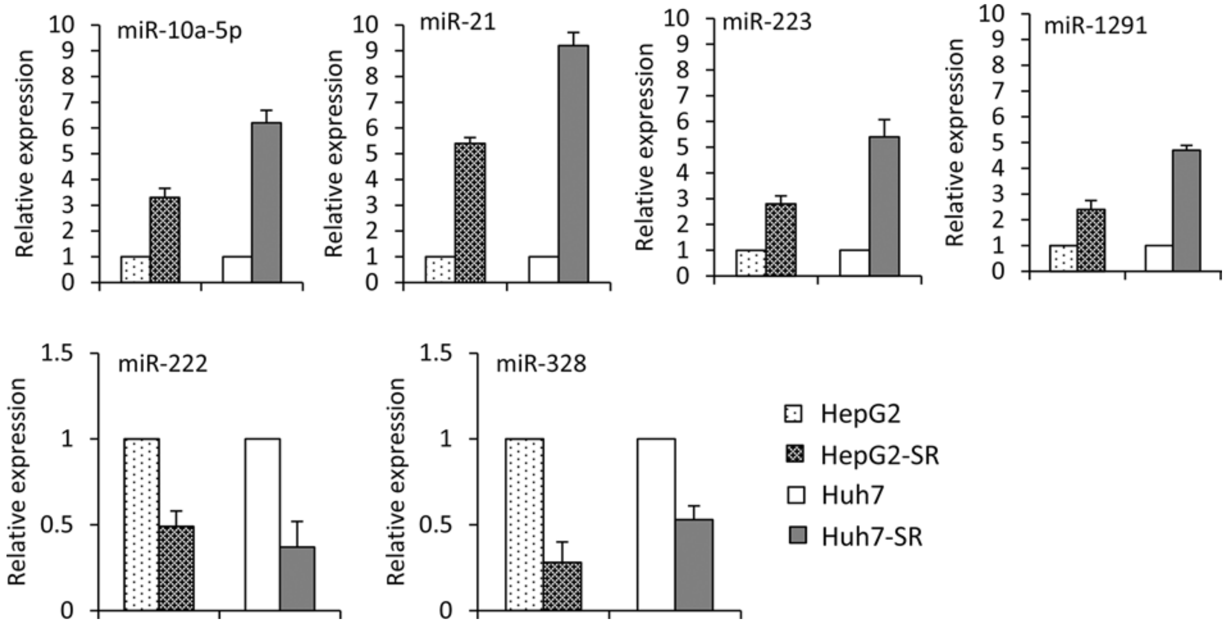

Supplementary Figure S4: The relative expression of selected miRNAs in HepG2, HepG2-SR, Huh7 and Huh7-SR cells. Twenty-five nanograms of total RNA from each cell line were subjected to real-time RT-PCR. The data were normalized to U6. The relative expression level of each miRNA in parental HepG2 or Huh7 cells was defined as 1.0.

Supplementary Table S1: Differentially expressed miRNAs in sorafenib-resistant vs. parental Huh7 cells

Upregulated miRNAs	Fold change	*P*-Value	Downregulated miRNAs	Fold change	*P*-Value
Let-7b	2.648	0.036	miR-17-5p	0.393	0.041
Let-7c	2.251	0.030	miR-18a	0.406	0.047
miR-10a-5p	5.143	<0.001	miR-133b	0.347	0.025
miR-10b-5p	3.024	0.019	miR-222	0.172	0.049
miR-34-a	3.032	0.042	miR-328	0.135	0.001
miR-21	7.278	<0.001	miR-548b-5p	0.465	0.018
miR-30a-3p	2.915	0.032	miR-675-5p	0.453	0.016
miR-195	2.837	0.029	miR-1290	0.451	0.023
miR-216a	2.252	0.014			
miR-219-1-3p	2.933	0.028			
miR-223	4.463	0.003			
miR-616	2.380	0.009			
miR-664	2.614	0.007			
miR-1260	2.362	0.025			
miR-1274a	2.729	0.017			
miR-1291	4.118	0.008			

NOTE: Sorafenib-resistant and parental Huh7 cells were subjected to a miRNA microarray. The expression level of each miRNA was measured, and fold change was calculated. Only those miRNA whose expression levels matched the 2-fold threshold are listed. Experiments were done in triplex. P value <0.05 was considered statistically significant.
